# Acute scrotum as a complication of Thiersch operation for rectal prolapse in a child

**DOI:** 10.1186/1471-2482-6-19

**Published:** 2006-12-28

**Authors:** Mohammad M Saleem, Hashem Al-Momani

**Affiliations:** 1 Department of Pediatric Surgery, Jordan University Hospital P.O. Box 13546, Amman 11942, Jordan

## Abstract

**Background:**

We report a case of acute scrotal condition that presented in a four year old male child one year after being treated for an idiopathic rectal prolapse utilizing Thiersch wire.

**Case presentation:**

The acute scrotum had resulted from spreading perianal infection due to erosion of the circlage wire. The condition was treated with antibiotics and removal of the wire. The child made an uneventful recovery.

**Conclusion:**

This case highlights that patients with Thiersch wire should be followed until the wire is removed. Awareness of anal lesions as a cause of acute scrotal conditions, and history and physical examination are emphasized.

## Background

Acute scrotum is a surgical emergency. Causes are usually intra scrotal conditions, of which torsion of the testis is the most important. Less common causes are extra scrotal in origin, namely idiopathic scrotal edema. Anal conditions are rare causes of acute scrotum. Diagnosis sometimes may be very difficult without surgical exploration. Careful history taking and physical examination including perianal region is emphasized.

## Case presentation

**Figure 1:**
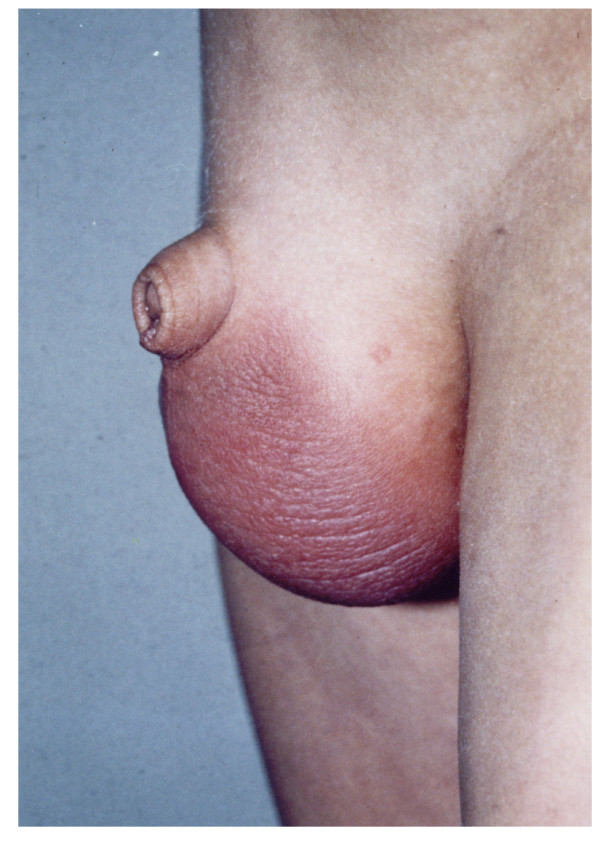
The child's scrotum. Note the redness and fullness of both scrotal compartments.

**Figure 2:**
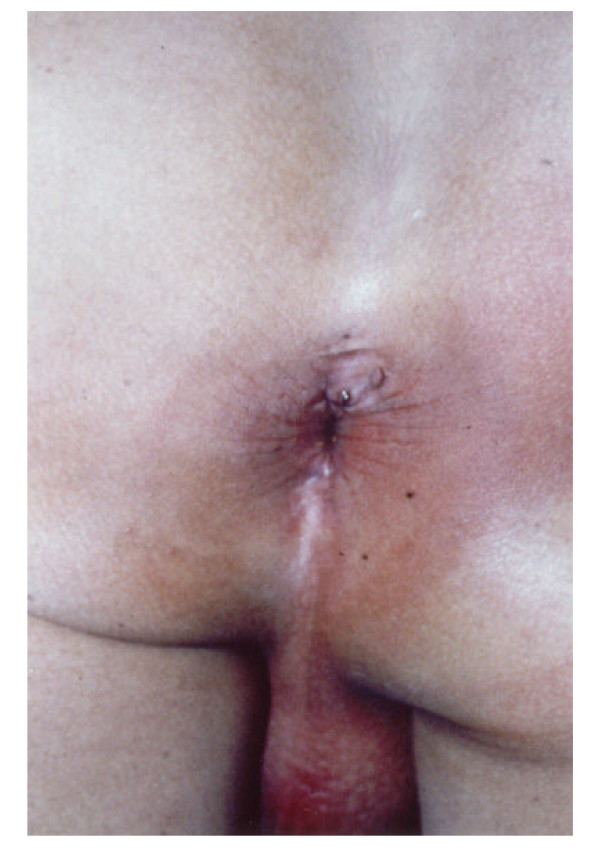
Thiersch's wire is shown eroding through the perianal skin.

B.S. a 4-year-old male child was admitted to the pediatric surgical ward at Jordan University Hospital because of two days history of swelling, redness and pain of his scrotum (Fig. 1). There was no history of trauma. The pain was moderate, not characteristic of torsion, and not the chief complaint. Oral temperature was 37.8°C and pulse 94/min. Physical examination revealed bilateral red swollen scrotum, moderately hot and tender allowing examination of the testes, which were in normal position and of equal size and not tender. There was a  small amount of fluid bilaterally detected on transillumination. The rest of the physical examination was unremarkable. Laboratory work up showed Hb. 10.5 gm/dl, Hct. 32%, WBC 12.7 × 10^3^/mm^3 ^and normal serum amylase. Ultrasound examination confirmed the equal sized testes, with normal echogenicity, the presence of small amount of fluid in the scrotum, and the edematous scrotum. On further examination of the scrotum, a streak of redness extended along the midline raphe to the perineum, where a visible wire through an eroded perianal skin was seen (Fig 2). The patient underwent a Thiersch wire insertion for a rectal prolapse one year before. The patient was started on broad-spectrum antibiotics (Ampicillin, Gentamycin and Metronidazol). It was decided that the Thiersch wire has to be removed in theatre under general anesthesia. While examination under anesthesia the perineum appeared healthy, without signs of inflammation, or suppuration but moderate amount of induration. Rectal exam was normal; there was moderate narrowing and fibrosis at the anal verge. After removal of the wire the tract was intact with minimal discharge, which cleared in few days following wire removal. The sinus tract was very short lived and closed without any problems thereafter.

Culture taken from the site of the wound grew streptococcus fecalis sensitive to the administered antibiotics. The scrotal swelling and redness subsided gradually. The child was discharged without any complications. Follow up was uneventful for both the scrotal redness and swelling as well as the rectal prolapse.

## Discussion

Common causes of acute scrotum at the age of four years are torsion of the testis or its appendix and incarcerated hernia. Less common causes are idiopathic scrotal edema and vasculitis (Henoch-Schonlein purpura). Rare causes are of anal pathology. Idiopathic scrotal edema was suggested by Nicholas et al to be of anal pathology origin [1]. Nour and MacKinnon reported ten cases out of 143 cases of acute scrotum were due to anal pathology [2].

The exact diagnosis of acute scrotum is very difficult in spite of available diagnostic means. The majority of cases require surgical exploration to rule out a testicular torsion. Careful history taking and physical examination can, as in this case, save the child from an operation.

Rectal prolapse in children is the result of self-limiting underlying disease, such as diarrhea, constipation, and weight loss [3]. Minor corrective procedures are injection of sclerosing agent [4,5], such as alcohol [6], phenol [7], or hypertonic saline[8] into the submucosa, cauterization of mucosa or anal encirclement according to the Thiersch-Ombredanne or its modification [8-10]. Each has its own advantages and recurrence rate. The advantages of the Thiersch operation are simplicity, effectiveness, and safety. The disadvantages are poor tolerance, rigidity, breakage and infection. Infection has always been described as local and superficial. As a matter of fact the principle of Ekehorn's operation for rectal prolapse is based on perirectal infection to prevent the rectum from prolapse by the formation of adhesions between the rectum and the surrounding structures [11]. Infection has also been reported after a Dacron prosthesis was used instead of wire, necessitating its removal [9]. Perineal clostridial myonecrosis has been described as a complication of Thiersch operation [12]. In the case we have reported the complication resulted from long standing wire acting as a hard foreign body that eroded the overlying skin resulting in perineal wound infection. Spreading of the infection may be related to the characteristics of the bacteria involved or because of shared lymphatic drainage. This case adds to the spectrum of the anal pathologies that may lead to scrotal infection. Awareness of such a complication, as well as close follow up of patients and a high degree of suspicion are the key to successful management.

## Conclusion

This case highlights that patients with Thiersch wire should be followed until the wire is removed. Awareness of anal lesions as a cause of acute scrotal conditions, and history and physical examination are emphasized

## Competing interests

The author(s) declare that they have no competing interests.

## Authors' contributions

Both authors, M.M.S, and H.M.A contributed equally to the case, and both have read and approved the manuscript

## Pre-publication history

The pre-publication history for this paper can be accessed here:
